# The COVID-19 patients' educational needs assessment questionnaire (COPENAQ): development and psychometrics

**DOI:** 10.1186/s12955-022-01922-0

**Published:** 2022-01-29

**Authors:** Reza Hajialibeigloo, Yaser Moradi, Hossein Habibzadeh, Rahim Baghaei, Vahid Alinejad, Mohammad Namazi Nia

**Affiliations:** 1 Patient Safety Research Center, Clinical Research Institute, School of Nursing and Midwifery, Urmia University of Medical Sciences, Urmia, Iran; 2 Biostatistics, Patient Safety Research Center, Clinical Research Institute, Urmia University of Medical Sciences, Urmia, Iran; 3 Department of Nursing, School of Nursing and Midwifery, Torbat Heydariyeh University of Medical Sciences, Torbat Heydariyeh, Iran

**Keywords:** Educational needs, COVID-19, Questionnaire, Psychometrics

## Abstract

**Background:**

Despite the importance of assessing COVID-19 patients' educational needs, there is currently no standard tool for a comprehensive assessment of these needs. The present study was aimed at developing a questionnaire to assess the educational needs of COVID-19 patients and determining its psychometric properties.

**Methods:**

This study was conducted using an exploratory sequential mixed methods design in 3 stages. At the first stage, patients' educational needs were explained and determined using conventional content analysis so that a total of 15 COVID-19 patients were first selected using purposive sampling and then interviewed. At the second stage, the questionnaire items were developed using the qualitative findings and a review of valid sources related to the study subject. At the third stage, the psychometric properties of the questionnaire were determined using internal consistency reliability and the face, content, and construct validities.

**Results:**

The mean content validity ratio and the content validity index were obtained to be 0.94 and 0.92, respectively. The internal consistency was evaluated using Cronbach's alpha, which was measured to be 0.97. Based on the exploratory factor analysis, the questionnaire was developed with 36 items in four subscales of "disease recognition and treatment follow-up", "prevention of infection transmission", "medication regimen", and "psychological and physiological needs". The results of confirmatory factor analysis also showed appropriateness and approval of the structural model obtained from the questionnaire.

**Conclusion:**

This questionnaire was found to have the necessary psychometric criteria (validity and reliability) so that it can be applied to assess the educational needs of COVID-19 patients and provide better and more effective patient education for them.

## Background

On 31 December 2020, an outbreak of atypical pneumonia due to a new coronavirus was reported in Wuhan, China [1]. According to the World Health Statistics, about 233,503,524 definite cases of COVID-19 and 4,777,503 deaths have been globally reported by 1 October 2021 [2].

Many people are discharged after a partial recovery from coronavirus disease 2019 (COVID-19) and spend their convalescence at home with their families. Many people need no hospitalization and their health condition is monitored by health professionals at home. During this period, patients and their family members should be educated about personal hygiene, basic measures of personal protection, and how to safely care for the patient and prevent the spread of infection to others [3].

The provision of patient education has become a major component of patient care [4]. Patient education is a heavy responsibility that lies with the members of the healthcare team, especially nurses. Patient education is in fact one of the essential components of nursing care leading to the promotion and restoration of health as well as the adaption to the disease complications [5]. Patient education strengthens health behaviors, changes unhealthy behaviors, and ensures community health [6, 7]. Patient education has many advantages for patients, families, physicians, and nurses. It also has a positive effect on adherence to treatment regimens and medical advice and helps to establish proper nurse-patient communication [8]. On the other hand, it leads to the proper use of health services, promotes the quality of care provision, and improves patient satisfaction, treatment adherence, and participation in self-care. Proper health education not only promotes patients' health behaviors but also reduces the financial burden of health care placed on them and society [9].

The educational needs assessment plays a key role in patient education [10]. The results of researches in this area have shown that the educational needs assessment causes people to have higher motivation for learning, improves learning, helps to provide patient education leading to functional behavior in patients [11, 12]. The determination of educational needs in order of priority is the first step in educational planning. Therefore, caregivers involved in the treatment-care process, especially nurses, should determine the patient's educational needs during the disease course and shortly thereafter [13, 14]. Numerous factors such as shorter hospital stay, early bed exit, readiness to recover, adaptation to illness, acceptance of new conditions, prevention of problems and complications caused by the illness, lifestyle changes, and treatment adherence based on the assessment and determination of patient educational needs are the prominent necessities of patient education [13, 15].

Despite the importance of needs assessment, there is currently no standard tool for a comprehensive assessment of COVID-19 patients' educational needs. On the other hand, recent studies have indicated that there are potential differences in the prioritization of health information and the understanding of care instructions between the patients and the health care providers [16]. Therefore, concerning the anxiety and stress caused by COVID-19 in the communities, there may be many questions in patients' minds that are different from the routine priorities of the health system so that the educational needs of these patients may be more important in some certain areas compared to other ones. The results of searching valid databases indicated that the available data were not sufficient to explain the educational needs of COVID-19 patients during the disease course. Given that methods such as literature review may not be sufficient alone in the development of quantitative tools, especially when a new structure is proposed, and that the researcher's knowledge may be limited in this area, the use of a qualitative approach based on experiences of patients in the area of existing and upcoming patients' educational needs is a fundamental item to be considered. Therefore, this study was conducted to develop and test the psychometrics of the COVID-19 Patients' Educational Needs Assessment Questionnaire (COPENAQ) as a quantitative and scientifically reliable tool for assessing the educational needs of COVID-19 patients.

## Method

This was an exploratory sequential mixed-methods study (qualitative-quantitative).

### Qualitative phase

#### Study design

First, the concepts of COVID-19 patients' educational needs and their dimensions were explained using conventional content analysis. Then the initial items of the questionnaire were developed based on both qualitative findings and a review of valid sources.

#### Study setting and participants

A total of 15 COVID-19 patients were recruited using purposive sampling. The study was conducted at Taleghani Hospital, Urmia, northwestern Iran. Inclusion criteria included the followings: (a) being literate, (b) willingness to participate in the study and share experiences. In order to achieve a wide range of experiences, participants were selected from outpatients and patients hospitalized in general wards who were going to be discharged.

#### Data collection

Data were collected by the first author (RH) and the corresponding author (YM) using semi-structured tele-interviews. The reason for the use of tele-interviews was to prevent any possibility of COVID-19 transmission. Participants were asked to describe their experiences and perceptions of their existing and future educational needs and the issues the health workers should provide patients with information on them. Based on the participants' responses, the interviewer led the interviews to a more in-depth review of their experiences about the study subject using probing questions such as "What do you mean?", "Can you explain more?", "Can you clarify what you mean?", "Why?", "How?". The researcher recorded all the interviews with the consent of the participants. Each interview lasted about 60 min. The interviews continued until the point at which data saturation was achieved.

#### Data analysis

Data were analyzed using the conventional content analysis approach in accordance with the proposed stages by Granheim and Landman [17]. At the end of each interview and note-taking, the recorded statements of the participants were first listened to by three authors (YM, RH, & RB), transcribed verbatim, and then typed in a Word document. Then, data analysis and concept extraction were conducted using MAXQDA software 10.0 R250412 (VERBI Software, 2019).

#### Rigor

After establishing intimate communication with the participants and gaining their trust, the researcher conducted the interviews in a friendly and comfortable atmosphere. Moreover, items including long-term engagement with data, spending enough time on data collection and analysis, participant review, and peer review were conducted to obtain a wide range of trustworthiness.

### Quantitative phase

The psychometric assessment of the questionnaire was conducted based on a methodological study. In this stage, psychometric properties of the questionnaire including quantitative and qualitative face validity, quantitative and qualitative content validity, construct validity, and reliability were examined.

#### Face validity assessment

The qualitative face validity was assessed by conducting interviews with a total of 10 patients with COVID-19, during which the level of difficulty in understanding the concepts, the degree of appropriateness and relevance, and the degree of ambiguity of each item were evaluated. In order to assess the quantitative face validity, the impact score of each item was calculated based on a 5-point Likert scale (Strongly important = 5, Quite a lot important = 4, Moderatly important = 3, Slightly important = 2, Not important at all = 1) and the following formula. If the impact score is more than 1.5, the item will be preserved and regarded as important by the target group.

$$Impact\;Score = Frequency\left( \% \right)*Importance$$

$$Frequency:\;Percentage\;of\;people\;who\;have\;checked\;the\;points\;of\;4\;and\;5.$$

$$Importance:\;People\;who\;have\;checked\;the\;points\;of\;4\;and\;5.$$

#### Content validity assessment

The content validity was evaluated using a quantitative approach [18] so that the content validity was evaluated based on the peer review process (expert panel checking). The validity assessment was made based on the viewpoints of 10 experts in the field of nursing. Finally, the content validity ratio (CVR) and the content validity index (CVI) were calculated.

The initial questionnaire was developed in 38 items and granted to a total of 10 experts to determine the CVR so that they were asked to rate each item based on a three-part spectrum including (a) it is necessary, (b) it is useful but not necessary, and (c) it is not necessary. Considering the number of panel experts (*n* = *10*), the minimum CVR was selected to be 0.62 based on the Lawshe table. Items with a CVR of greater than 0.62 were considered significant (*p* < 0.05) and preserved.

The CVI was examined using Waltz and Bausell's approach [19]. The appropriateness, clarity, ambiguity, and relevance of each item to the study objective were considered as the main CVI assessment points by the experts [20]. So a number of 10 experts (other than the ones who had examined the CVR) separately examined the three criteria of simplicity, relevance, and clarity for each item based on a 4-point Likert scale. The CVI for each item was calculated by dividing the number of experts who had checked the scores 3 and 4 by the total number of experts. Hyrkäs et al. [21] recommended the scores of 0.79 for accepting items based on CVI. Moreover, if the item score ranges between 0.7 and 0.79, it will need to be revised.

#### Construct validity assessment

The construct validity of COPENAQ was examined in a cross-sectional study using exploratory factor analysis (EFA) and confirmatory factor analysis (CFA). Concerning the need for collecting information for the construct validity assessment, a number of 3–5 samples per item or 10–20 samples per construct is sufficient to perform EFA based on the recommendations cited in scientific sources [22]. After developing the final version of the questionnaire, a number of 5 samples were considered for each item to ensure the sufficiency of sample size. In the construct validity assessment of the COPENAQ, a total of 36 items remained and 2 items were removed. Considering the probability of 10% sample attrition, a total of 200 outpatients and inpatients with COVID-19 admitted to general wards of COVID treatment centers were recruited using convenience sampling.

In this study, the Kaiser–Meyer–Olkin (KMO) measure of sampling adequacy and Bartlett's test of sphericity were utilized to evaluate the appropriateness of data for performing EFA (Table 1).

**Table 1:** The results of the Kaiser–Meyer–Olkin (KMO) measure and the Bartlett's test of spherocity

Kaiser–Meyer–Olkin measure of sampling adequacy		0.919
Bartlett's test of sphericity	Approx. chi-square	7990.503
	df	946
	Sig	0.000

In the KMO measure of sampling adequacy, the KMO value varies between 0 and 1. A value of 0 shows that the correlations matrix is diffused and the factor analysis is not usable. In contrast, values close to 1 indicate the relative compactness of the correlations matrix so that factor analysis can be used to enable the separation of factors. The KMO values of 0.5–0.7, 0.7–0.8, 0.8–0.9, and > 0.9 indicate mediocre, middling, meritorious, and marvelous factor analysis, respectively [23].

Bartlett's test of sphericity measures the null hypothesis as it assesses whether the correlations matrix is significantly different from an identity matrix. In order to use factor analysis, the variables must have a correlation and all correlation coefficients are equal to 0 when the matrix is significant [24].

The EFA was conducted using principal component analysis (PCA) and varimax rotation. In order to preserve the item in the EFA, the factor loading was considered to be 0.4 at least [25]. After conducting the EFA, the CFA was applied to analyze the components, factors, and their markers. The CFA answers the question "Do the selected markers represent or fit the factors?".

#### Reliability assessment

The reliability of the questionnaire was assessed based on the internal consistency method with a sample of 20 eligible patients with COVID-19 as the Cronbach's alpha coefficient was calculated for each subscale and the whole questionnaire as well.

#### Ethical considerations

The study protocol was approved by the Regional Committee of Medical and Health Research Ethics (Ethics ID: IR.UMSU.REC.1399.213). Prior to the beginning of the study, the study objectives were explained to the participants and the written informed consent to conduct, record, and transcribe interviews was obtained from all of them. Participants were also assured that their personal data would be regarded as completely confidential and they would be able to access the results.

## Results

### Qualitative phase: development of the questionnaire

Out of 15 patients who participated in the qualitative phase of the study, 8 were male and 7 were female. They were in the 22–51 age group and their educational status ranged from literacy to higher education. Out of 15 participants recruited in this phase, 9 referred to the hospital clinic as outpatients and 6 were previously hospitalized in general wards and going to be discharged. Most participants were suffering from typical symptoms of COVID-19 including fever, cough, body aches, and shortness of breath.

Data analysis in the qualitative phase led to the identification of patients' educational needs in eight domains, including disease recognition and treatment follow-up, personal hygiene, prevention of infection transmission, medication regimen, nutrition and excretion, sleep and daily activities, mental health issues, and sexual activity. An extensive literature review was then conducted.

The above two steps were combined and a total of 38 practical items were designed using a 5-point Likert scale to assess the educational needs of COVID-19 patients.

### Quantitative phase: psychometric properties

In the process of face validity assessment, the results showed that the impact score of all items of the initial questionnaire was more than 1.5, which was the indicator of the appropriateness of all items for entering the content validity assessment.

Content validity was assessed by calculating CVI and CVR. The CVR numerical value of all items was shown to be more than 0.62. This value was considered significant (*p* < 0.05) and still led to the preservation of all items. However, 2 items were omitted at the CVI assessment stage. The sum of CVRs and CVIs was calculated to be 0.94 and 0.92, respectively.

In the next step, construct validity of the 36-item questionnaire was conducted using EFA. At this stage of the study, a number of 200 patients with a mean age of 36.5 ± 9.79, whose demographic characteristics are reported in Table 2, completed the questionnaire.

**Table 2:** Demographic characteristics of participants (*n* = 200)

Characteristics	Categories	n (%)
Gender	Male	75 (37.5)
	Female	125 (62.5)
Marital status	Married	159 (79.5)
	Single	41 (20.5)
Education	Primary	31 (15.5)
	Secondary	100 (50)
	Tertiary	69 (34.5)
Admission	Outpatient	90 (45)
	Inpatient	110 (55)

**Fig. 1:**
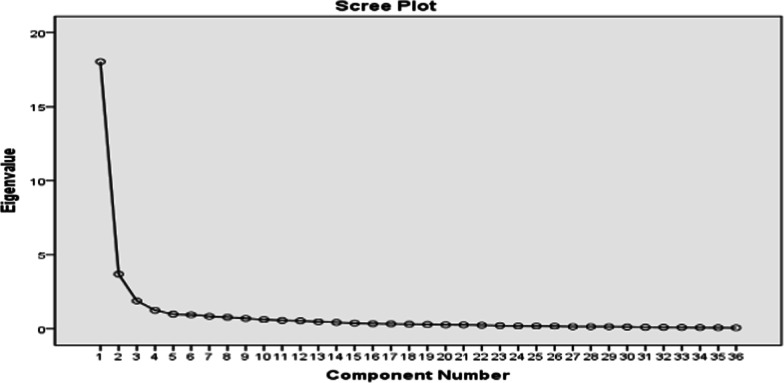
Scree plot of the exploratory factor analysis of COPENAQ

Considering the results of EFA, eigenvalues of greater than 1, and the slope of scree plot (Fig. 1), four factors were extracted all of which had a predictive power of 69.076% for the total educational needs of COVID-19 patients (Table 3, Fig. 1).

**Table 3:** Total variance obtained based on the exploratory factor analysis (EFA)

Component	Initial eigenvalues			Extraction sums of squared loadings			Rotation sums of squared loadings		
	Cumulative %	% of variance	Total	Cumulative %	% of variance	Total	Cumulative %	% of variance	Total
1	50.136	50.136	18.049	50.136	50.136	18.049	25.532	25.532	9.191
2	60.394	10.258	3.693	60.394	10.258	3.693	49.499	23.967	8.628
3	65.624	5.230	1.883	65.624	5.230	1.883	61.349	11.851	4.266
4	69.076	3.452	1.243	69.076	3.452	1.243	69.076	7.726	2.782

Based on the results of EFA with varimax rotation, a 36-item questionnaire was developed in 4 subscales of "disease recognition and treatment follow-up" (5 items) "prevention of infection transmission" (12 items), "medication regimen" (5 items), and "psychological and physiological needs" (14 items) (Table 4).

**Table 4:** The results of exploratory factor analysis (EFA) with varimax rotation

Item no	Factors			
	Disease recognition and treatment follow-up	Prevention of infection transmission	Medication regimen	Psychological and physiological needs
1	0.648			
2	0.679			
3	0.739			
4	0.651			
5	0.655			
1		0.438		
2		0.764		
3		0.787		
4		0.822		
5		0.837		
6		0.792		
7		0.674		
8		0.596		
9		0.542		
10		00.693		
11		0.428		
12		0.674		
1			0.558	
2			0.605	
3			0.570	
4			0.531	
5			0.492	
1				0.481
2				0.741
3				0.810
4				0.703
5				0.671
6				0.683
7				0.718
8				0.711
9				0.698
10				0.748
11				0.702
12				0.814
13				0.854
14				0.831

**Fig. 2:**
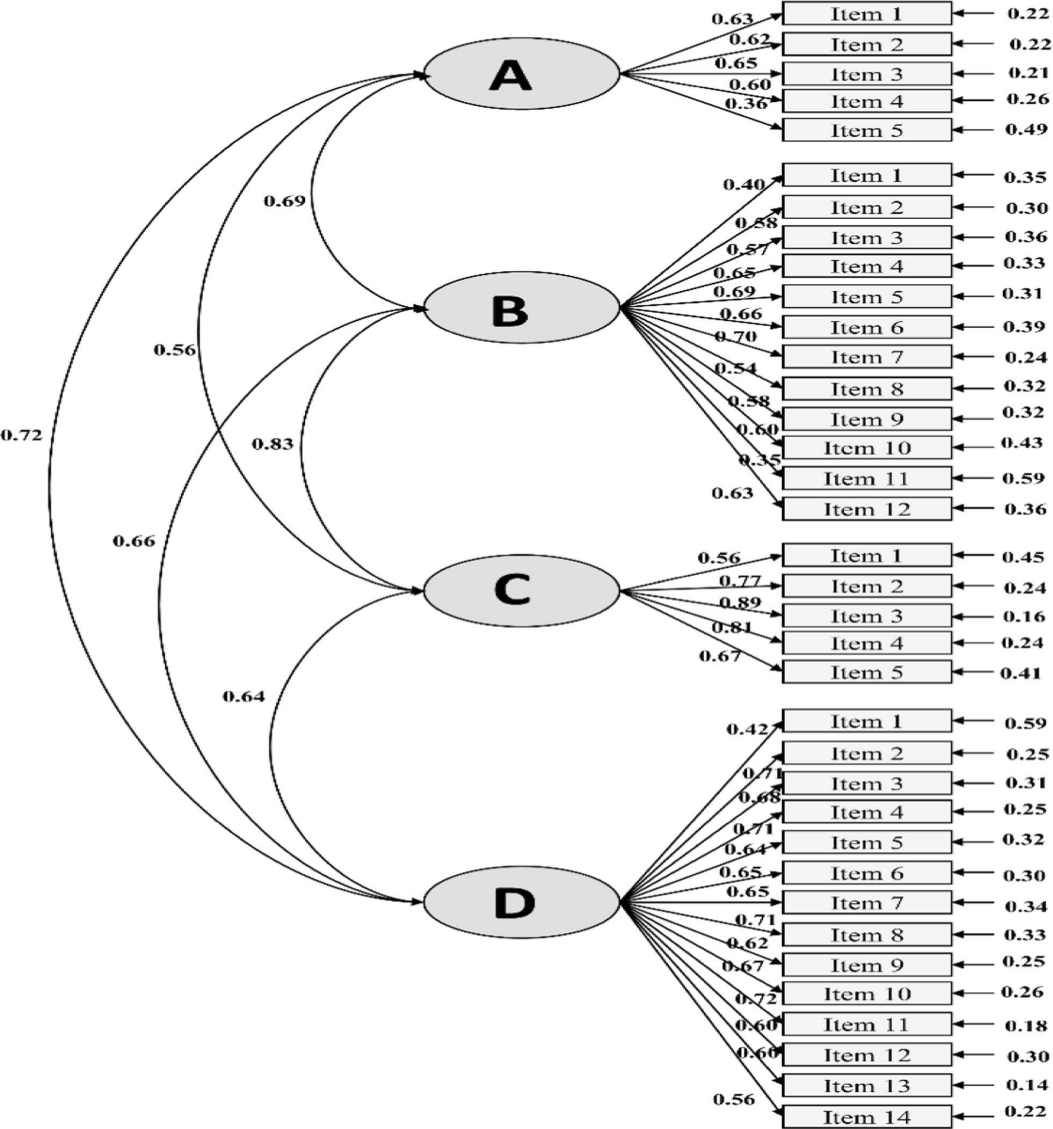
Results from confirmatory factor analysis. **A** Disease recognition and treatment follow-up. **B** Prevention of infection transmission. **C** Medication regimen. **D** Psychological and physiological needs. Root mean square error of approximation (RMSEA) = 0.12, 90 percent confidence interval for RMSEA = (0.11; 0.12). *P* value for test of close fit (RMSEA < 0.05) = 0.0001

In the next step, the CFA was used to confirm the structure of the questionnaire. The results of the CFA and the root mean square error of approximation (RMSEA) criterion indicated the approval and appropriateness of the structural model obtained from the COPENAQ (Fig. 2).

After the conduct of CFA, the internal consistency of the questionnaire was assessed in a sample size of 200 patients with COVID-19 using Cronbach's alpha. Cronbach's alpha coefficient was calculated to be 0.97 and 0.85–0.96 for the whole questionnaire and each subscale, respectively (Table 5).

**Table 5:** Cronbach’s alpha calculated for the whole questionnaire (COPENAQ) and its subscales

Factors	Cronbach’s α coefficient
Disease recognition and treatment follow-up	0.85
Prevention of infection transmission	0.94
Medication regimen	0.93
Psychological and physiological needs	0.96
Total	0.97

### Scoring method

All items of the COPENAQ are scored on a 5-point Likert scale from "1 = Not Important At All" to "5 = Extremely Important". Moreover, the score of each subscale is converted to an index of 100 based on the following formulas. Finally, the scores obtained for each subscale are classified into four levels of low (0–25), medium (26–50), high (51–75), and very high (76–100) to identify the level of patients' educational needs.

$$Disease\;recognition\;and\;treatment\;follow{-}up = \frac{Subscale\;score - 5}{{20}} \times 100$$

$${\text{Prevention}}\;{\text{of}}\;{\text{infection}}\;{\text{transmission}} = \frac{Subscale\;score - 12}{{48}} \times 100$$

$${\text{Medication}}\;{\text{regimen}} = \frac{Subscale\;score - 5}{{20}} \times 100$$

$${\text{Psychological}}\;{\text{and}}\;{\text{physiological}}\;{\text{needs}} = \frac{Subscale\;score - 14}{{56}} \times 100$$

### Description of the scale

The COPENAQ is a valid 36-item questionnaire developed in 4 subscales and measures the importance of patients' educational needs at 4 levels from ''slightly important"" to " highly important"". After the examination of psychometric properties, the final version of the questionnaire was developed (Table 6).

**Table 6:** Items of the COPENAQ

Subscale	No	Item	Not important at all	Slightly important	Moderately important	Very important	Extremely important
Disease recognition and treatment follow-up	1	What symptoms may be associated with my disease?					
	2	What complications can happen to me following my disease?					
	3	What should I do if the disease symptoms (e.g., cough, shortness of breath, headache) worsen?					
	4	In what circumstances should I refer to a doctor/hospital again?					
	5	How is the disease transmitted to those around me?					
Prevention of infection transmission	6	How should I isolate myself to prevent the disease transmission to my family members?					
	7	How long is the minimum isolation period after discharge?					
	8	When should I definitely wash or sanitize my hands?					
	9	What is the exact method of handwashing to kill germs and viruses and how long should it be?					
	10	Which hand sanitizer is better for washing or sanitizing my hands?					
	11	When and how should I use gloves?					
	12	When should I use a face mask?					
	13	What kind of mask should I use?					
	14	How should I use a face mask?					
	15	What can I do to prevent the disease transmission in shared spaces of the house (e.g., bathroom, toilet)?					
	16	What should I do to prevent the disease transmission when coughing and sneezing?					
	17	What should I do to disinfect my resting place?					
Medication regimen	18	How should I take my medications?					
	19	When should I take my medications?					
	20	What is the reason for prescribing each of my medications?					
	21	What are the adverse effects of my medications and what should I do when they occur?					
	22	What should I do if I miss a dose of my medication?					
Psychological and physiological needs	23	What foods should I consume and what foods should I avoid?					
	24	Do I need to take certain dietary supplements (e.g. vitamins or multivitamins)?					
	25	How should I prepare the food I want to consume?					
	26	How should I deal with problems affecting my diet (e.g., nausea, vomiting, diarrhea, pain)?					
	27	How much should I rest during the day?					
	28	When can I return to work?					
	29	Does the treatment of this disease require any specific activity restrictions?					
	30	What should I do if I do not get regular sleep?					
	31	How can I control my restlessness and anxiety?					
	32	What can I do to overcome the sense of nostalgia during the isolation period?					
	33	How can I avoid aggression?					
	34	When can I have sex after discharge?					
	35	What important health points should I stick to in my sexual relationship?					
	36	Is my sexual partner (my spouse) at risk of contracting the disease through sexual intercourse?					

## Discussion

This was a cross-sectional study aimed at developing a questionnaire to assess the educational needs of COVID-19 patients and determining its psychometric properties. In a literature review conducted by the authors, it was found that some studies have been conducted on the needs assessment tools in Iran and other countries around the world. Some of these tools are developed only for specific diseases, especially chronic ones. Based on the investigation conducted by the authors, no questionnaire has been still developed to assess the educational needs of patients with acute and infectious diseases, especially COVID-19, at such a critical time when the focus of policymakers in all countries is on this issue. In order to fill the existing gap in this area, the present study was conducted using an exploratory sequential mixed methods design to develop a tool named " COPENAQ" for assessing the educational needs of patients with COVID-19. The validity of this tool was also assessed using a quantitative approach.

The qualitative phase of the present study maximizes the likelihood that the COPENAQ addresses the important needs of patients and has a high face and content validity. In addition, the extensive literature review opened up a unique opportunity for COPENAQ developers to address the potential needs of patients in various dimensions. Quantitative findings of this study indicated that the COPENAQ, which was developed in 4 subscales and 36 items, has good psychometric properties.

Bensing (2001) emphasized the need to adjust educational support materials based on the needs and characteristics of patients. Needs assessment tools can be applied in the area of patient education and may affect the patient's adherence to treatment and other therapeutic outcomes [26].

The first subscale of the COPENAQ is "disease recognition and treatment follow-up". This subscale consists of 5 items and assesses the patient's educational needs in the areas including symptoms and complications of the disease, necessary measures in case of serious and severe symptoms, time of the hospital/doctor visit, and methods of disease transmission. The lung information needs questionnaire (LINQ), which has been developed to assess the educational needs of patients with respiratory conditions, can be utilized to compare the COPENAQ with other similar questionnaires. There are four questions in the "disease recognition" factor of this tool that examine the level of patient information about the disease name, its impact on the respiratory tract, and the future health condition of the patient. Another factor of LINQ is called "self-management", which includes 2 questions evaluating patient information about the necessary measures in case of disease exacerbation and emergency medical services (EMS) contact time [27]. The two above factors of the LINQ significantly overlap with the "disease recognition and treatment follow-up" factor of the COPENAQ.

The COPENAQ not only reflects the information needs of patients but also covers the existing information and concepts at the heart of COVID-19 management guidelines. One of the most important concepts is "prevention of infection transmission", which includes items on the ways to isolate oneself during convalescence and the effect of hygiene observation on preventing disease transmission among family members [28]. This factor of the COPENAQ is somewhat unique since the existing questionnaires are developed to assess the needs of patients with chronic and non-communicable diseases while the COPENAQ is developed to measure the needs of patients with an acute and contagious disease. This factor is very important since the patient's lack of awareness about the observance of health protocols can easily and directly threaten the patient's family members and the community as well. Moreover, viral mutations caused the COVID-19 to be highly contagious [29].

The third factor of the COPENAQ examines the patient's educational needs in the area of "medication regimen". This factor can be seen in most of the questionnaires developed for assessing patients' educational needs [27, 30]. Knowledge about the name of medications, the cause and method of using medications, and their adverse effects can be very effective in improving patients' self-efficacy and adherence to medication regimens [31].

The last factor of the COPENAQ is "psychological and physiological needs". The results of studies conducted on psychological problems of COVID-19 patients emphasize the importance of early implementation of supportive interventions based on the educational and care needs of these patients [27]. Therefore, the use of COPENAQ can be very helpful in this regard. This factor of the COPENAQ examines the educational needs of COVID-19 patients in areas including physical activity and rest, nutrition, sexual activity, and mental health issues, two of which were mentioned in the LINQ under the headings of "exercise" (4 items) and "diet" (1 item) [27]. Moreover, in the Patient Learning Needs Scale (PLNS), which is a tool to assess the educational needs of patients at the time of discharge, psychological issues are mentioned in a factor called "feelings related to condition" [30].

COPENAQ is developed and validated to assess the educational needs of patients with COVID-19 in the Iranian healthcare system. However, considering the high validity and reliability of the questionnaire, it can be used not only in Iran but also in the healthcare system of other countries. To use this questionnaire in other contexts, it is recommended to first translate and then validate it for the target population.

## Conclusion

The COPENAQ is a valid (*S-CVR* = 0.94*, S-CVI* = 0.92) and reliable (*α* = 0.97) 36-item questionnaire for assessing the educational needs of patients with COVID-19. This questionnaire is made up of 4 subscales, including "disease recognition and treatment follow-up", "prevention of infection transmission", "medication regimen", and "psychological and physiological needs", which predict 69.076% of the variance of the total educational needs of COVID-19 patients. Moreover, all items of this questionnaire are scored on a 5-point Likert scale from "1 = Not Important At All" to "5 = Extremely Important".

## Data Availability

The datasets used and/or analyzed the current study are available from the corresponding author on reasonable request.

## Abbreviations

COPENAQ: COVID-19 patients' educational needs assessment questionnaire
WHO: World Health Organization
*COVID-19*: Coronavirus disease 2019
LINQ: Information needs questionnaire
PLNS: Patient learning needs scale
